# Protective effect of hydroxysafflor yellow A on cyclosporin A-induced renal oxidative stress in vitro and in vivo

**DOI:** 10.1590/acb370305

**Published:** 2022-06-15

**Authors:** Jiyuan Wang, Yu Chen

**Affiliations:** 1 MSc. Second Military Medical University – Shanghai ChangZheng Hospital – Department of Organ Transplantation – Shanghai, China.

**Keywords:** Cyclosporine, Oxidative Stress, Apoptosis

## Abstract

**Purpose:**

To explore the mechanism and investigate the protective effect of hydroxysafflor yellow A (HSYA) on renal oxidative stress, which cyclosporine A (CsA) induces.

**Methods:**

HK-2 cells were treated with CsA to get CsA-induced oxidative stress. The effects on oxidative stress and apoptosis of HK-2 cells were detected. The contents of SOD, MDA, GSH-Px, ROS, and CAT in serum were measured, and the expression of apoptosis-related proteins was detected by western blot. Then, established the renal oxidative stress injury rats to verify the efficacy of HSYA.

**Results:**

HSYA could reduce the ROS and MDA contents induced by CsA. Compared with the CsA group, the activities of SOD, CAT, and GSH-Px increased significantly when treated with HSYA. HSYA could inhibit CsA-induced apoptosis in HK-2 cells, and promote the protein of Bcl-2 and inhibit the expression of Bax. Animal experiments showed that HSYA could reduce CsA-induced renal cell injury by reducing glomerular cell vacuoles and inflammatory factors in tissues. It also decreased serum creatinine (Crea) and blood urea nitrogen, increased Crea clearance significantly.

**Conclusions:**

HSYA could significantly improve the antioxidant capacity of the kidney cells and inhibit cell apoptosis, thereby effectively ameliorating CsA-induced oxidative stress *in vitro* and *in vivo*.

## Introduction

As a commonly used immunosuppressant after kidney transplantation, one of the side effects of cyclosporine A (CsA) is the occurrence of chronic allograft nephropathy (CAN). This is the main reason why the patient re-dialysis after transplantation^1^. Currently, the mechanism of CAN caused by CsA is not precise. The existing findings suggest that the increase of reactive oxidative metabolites, and the decrease of renal antioxidant capacity caused by CsA are critical factors of CAN^2^. And CsA can directly cause lipid peroxidation in the endothelial cell membrane, induce oxidative stress in the cell, and enhance the production of oxygen-free radicals^2^. Furthermore, oxidative stress is closely related to the development of CAN. Pathological manifestations are the gradual development of glomerulosclerosis and interstitial fibrosis, accompanied by inflammatory infiltration and atrophy of renal cells^3^. The clinical manifestations are progressive deterioration of renal function, hypertension, proteinuria and so on. Reactive oxygen species (ROS) are inevitably produced during ischemia-reperfusion during organ transplantation and are characterized by oxidative stress that induces tissue damage immediately after renal transplantation^4^. In addition, oxidative stress is involved in the renal injury during ischemia-reperfusion. This oxidative imbalance may trigger an inflammatory response within the transplanted organ leading to tissue damage and graft dysfunction if the clearance capacity of the kidney is not sufficient to produce excess ROS^5^. It has also been confirmed in animal models^6^.

It has been observed that the addition of antioxidants (e.g., vitamins E and C) to CsA-treated cells prevents CsA cytotoxicity and lipid peroxidation^7^. This implies that the application of appropriate antioxidants can ameliorate CsA-induced renal function and histological damage. Hydroxysafflor yellow A (HSYA) is a natural compound from *Carthamus tinctorius L*. It has a broad and effective pharmacological activity^8^. Modern pharmacological experiments have proved that red anthocyanin has antithrombotic, antioxidation, anti-inflammatory, anticancer effects, and has low toxic and side effects, and protective effect on ischemia-reperfusion injury^9^. Recent research showed that HSYA could against acute kidney injury^10^. Some studies indicate that HSYA reduces the oxidative stress level in a rat renal ischemia-reperfusion injury model. Thus, it can be inferred that’s the reason for alleviating renal ischemia-reperfusion injury^11^.

Therefore, the HSYA might protect against CsA-induced renal oxidative stress. However, whether HSYA could alleviate CsA-induced chronic kidney injury in rats by affecting oxidative stress has not been systematically reported. This study will systematically explain the effect and mechanism of HSYA on chronic renal transplantation from two levels of cells and animal models. This experiment provides the experimental basis for further development and application of HSYA.

## Methods

### Materials

Hydroxysafflor yellow A (98.05%, HY-N0567, MedChemExpree, USA), CsA (99.85%, HY-B0579, MedChemExpree, USA), N-acetyl-L-cysteine (NAC, A7250, Sigma-Aldrich, USA). Cell line’s basal medium and nonkeratinocyte culture medium (K-SFM, 17005-042, Invitrogen, Thermo, MA, USA). Radioimmunoprecipitation assay (RIPA) lysis and extraction buffer (P0013B, Beyotime, Shanghai, China), rabbit antihuman Bax primary antibody (5023, Cell Signaling Technology, Inc. USA), and horseradish oxidase-linked goat antirabbit secondary antibody (7074, Cell Signaling Technology, Inc. USA). Super Signal Enhanced Chemiluminescence Kit (34094, Thermo, USA). The ones bought from Abcam (Cambridge, UK) are Rabbit antihuman Bcl-2 antibody (ab194583), ?-actin primary antibody (ab227387), anti-nuclear factor-?B (anti-NF-κB) p65 antibody (cab16502), and Schiff staining kit (ab150680). Superoxide dismutase (A001-3), malondialdehyde (A003-1), glutathione peroxidase (A005), ROS (E004), and peroxide hydrogenase (A007-1-1) detection kits were purchased from NJJC Bio (Nanjing, China). Hematoxylin and eosin (H&E) staining kit (C0105, Beyotime Biotechnology (Shanghai, China). Other reagents are of analytical grade.

### Cell culture

HK-2 cells were from Ruyao Biotechnology (Zhejiang, China). Provided BPE (0.05 mg·mL^–1^) and EGF (5 ng·mL^–1^)with a K-SFM kit, both are necessary for cell line growth. Culture conditions: 95% O_2_, 5% CO_2_, 37.0 °C and humidity 70–80%.

### Methylthiazolyldiphenyl-tetrazolium bromide (MTT) determination

The HK-2 cells in the logarithmic growth phase were seeded in a 96-well plate (5 × 10^3^ cells per well), and added different concentrations of HSYA (0, 20, 40, 80, and 160 μmol·L^–1^) mixed with CsA (2 μmol·L^–1^) for 24 h. Then added 20 μmol·L^–1^ MTT (5 mg·mL^–1^) to each well and incubated at 37 °C for 4 h. Discarded the supernatant and added 200 μL DMSO. After shaking for 10 min, the wavelength of 560 nm was selected to measure the absorbance value of each hole on the enzyme-linked immunosorbent monitor.

### Oxidative stress detection

In order to detect oxidative stress, HK-2 cells (2 × 10^4^ cells·well^–1^) were divided into the following seven groups according to the variable HSYA: the control group (equal volume of normal saline), 40 μmol·L^–1^ HSYA, 2 μmol·L^–1^ CsA, 20 μmol·L^–1^ HSYA+2 μmol·L^–1^ CsA, 40 μmol·L^–1^ HSYA+2 μmol·L^–1^ CsA, 80 μmol·L^–1^ HSYA+2 μmol·L^–1^ CsA and 5 mmol·L^–1^ N-acetyl-L-cysteine (NAC). After culturing in an incubator for 48 h (37 °C, 5% CO_2_), washed the cells with PBS, added 1 mL of saline, placed them in a test tube, and lysed the cells with ultrasound. Detected the superoxide dismutase (SOD), malondialdehyde (MDA), glutathione peroxidase (GSH-Px), reactive oxygen species (ROS), and catalase (CAT) in cell lysates (centrifuged at 12000 × g, 4 °C for 10 min) by the test kits. Three parallel experiments were set up for the above experiments.

### Apoptosis

HK-2 cells were added to different drugs and cultured for 48 h, collect the cells and detect the level of apoptosis according to Annexin V-FITC Apoptosis Detection Kit. Washed cells with PBS twice, resuspended cells in 1 × PBS buffer, then added annexin V labeled with fluorescein isothiocyanate and PI each 5 μL. Analyzed apoptosis by flow cytometry after incubating HK-2 cells for 15 min at room temperature.

### Western blot

After being washed three times with precooled PBS, the cells were lysed slowly on ice with moderate RIPA. Collected the cell fragments and lysates and obtain the supernatant (the total protein mixture) by centrifugation (13.000 × g for 12 min at 4 °C). It uses bicinchoninic acid (BCA) for the protein concentration assay. The sample (40 ?g) was loaded into the 10% SDS-PAGE gel. The protein was transferred to the PVDF membrane after 2 h of SDS-PAGE electrophoresis. Subsequently, it was blocked with 5% skim milk at room temperature for 2 h, then added primary antibodies, including rabbit antihuman Bax (diluted 1:1500), anti-?-actin antibody (diluted 1:2000), and Bcl-2 (1:1000 dilution). The antibody was kept at 4 °C overnight, and washed the membrane with 1 × TBS (0.1% Tween-20) three times. According to the instructions, diluted HRP-labeled goat antirabbit IgG as the secondary antibody. Two hours for incubation at room temperature. Super Signal Enhanced Chemiluminescence Kit for Protein coloration and gel imaging system (UVP, USA) for protein banding imaging. The protein expression level was measured by the intensity of gray value with Image J software.

### Chronic allograft nephropathy (CAN) animal model

Male SD rats (n = 18, 7 weeks old, clean grade), 200–250 g, purchased from Shanghai Slack Experimental Animal Company, and raised in the Shanghai ChangZheng Hospital. The temperature was kept at 23 ± 1.5 °C. Humidity is 40–60%, alternating light and dark every 12/12 h, free drinking and food. The feed is a complete nutrient pellet feed. Divided rats randomly into three groups: (1) Control group: inject 0.9% NaCl (4 mL·[kg·d]^–1^), (2) positive control, HSYA (40 mg·[kg·d]^–1^), (3) CsA group: 0.9% NaCl (4 mL·[kg·d]^–1^) + CsA (30 mg·[kg·d]^–1^) dissolved in purified water) subcutaneous injection. (3) CsA+HSYA group: HSYA (10 mg·[kg·d]^–1^ dissolved in purified water) + CsA (20 mg·[kg·d]^–1^) subcutaneous injection, (4) HSYA (20 mg·[kg·d]^–1^ + 20 mg·[kg·d]^–1^ CsA) group, (5) HSYA (40 mg·[kg·d]^–1^ + 20 mg·[kg·d]^–1^ CsA) group, (6) HSYA (60 mg·[kg·d]^–1^ + 20 mg·[kg·d]^–1^ CsA) group, (7) NAC group (5 mg·[kg·d]^–1^). Each group was administered for 21 days. The experimental protocol gets the approval of the experimental animal ethics committee of Shanghai ChangZheng Hospital (the license certificate is included in the supplementary materials).

### Renal function index test

On the 22nd day, collected the three groups of rats’ urine within 24 h (each group is 6). Rats were anesthetized with chloral hydrate, 3–4 mL blood samples were collected through the abdominal aorta, and centrifuged at 3000 r·min^–1^ for 30 min to prepare serum. Measured serum creatinine (Crea) and serum urea nitrogen (BUN) levels, and calculated creatinine clearance (Ccr); Ccr = (Ucr/Scr·V) (Ucr: urine creatinine, μmol·L^–1^), Scr: serum creatinine (μmol·L^–1^), V: urine volume (mL·min^–1^).

### Histopathological examination

On day 22, the three groups of rats (each group is n = 6) were sacrificed by cervical termination, and kidneys were removed and fixed with 5% formaldehyde for 48 h at 4 °C. Tissue paraffin blocks were prepared and sectioned (5 μm). Histological study: Hematoxylin staining was performed on rats’ kidney tissue sections after deparaffinization and rehydration with the help of a H&E staining kit. Periodic acid – Schiff (PAS) staining was used: immersed the slides in the periodic acid solution for 10 min. Rinsed the slides with distilled water four times, and then soaked in Schiff solution at 20 °C for 30 min. Rinse the slide with a gentle jet of water. Three minutes were needed for the slides stained with hematoxylin and 5 min in running water for rinsing. Finally, the slides washed with distilled water are dehydrated by gradient alcohol. Immunohistochemistry was used to detect the expression of NF-κB in kidney tissue. In brief, after deparaffinization, paraffin section tissue was incubated with anti-NF-κB p65 antibody (diluted 1:1000) at 4 °C overnight, and horseradish peroxidase-conjugated goat antirabbit. The secondary antibody was kept at room temperature for 2 h. After 3,3’-diaminobenzidine (DAB) staining and antifading oil fixing, observed on an optical microscope and took pictures.

### Statistical analysis

All data were shown as the mean ± standard error of the mean. Using SPSS 22.0 software (USA) measured two groups difference according to the Student’s t-test. Differences between multiple groups were analyzed by one-way analysis of variance, and the post hoc test was done by Tukey’s test. Statistical p < 0.05 represents that the difference between the control groups is significant.

## Results

### HSYA relieves CsA-induced oxidative stress in HK-2 cells

**Figure 1:**
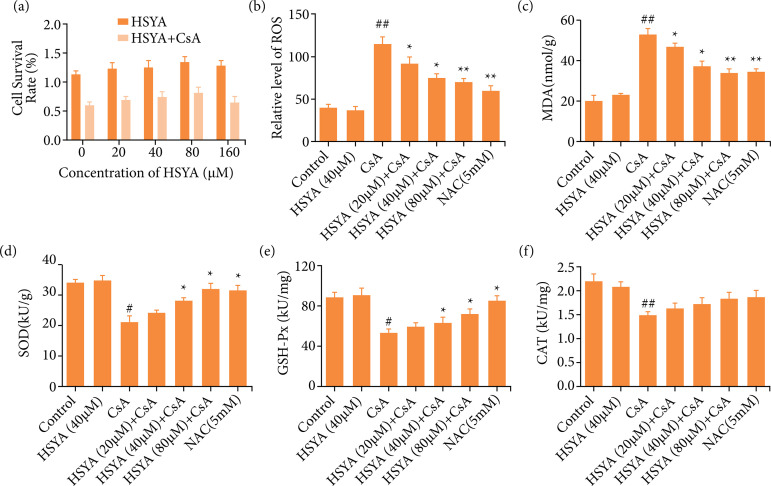
The effects of HSYA at different concentrations on HK-2 cell viability, oxidative stress-related factors and antioxidant enzyme activities. **(a)** MTT assay for measuring the survival rate of cells to assess the effect of HSYA on HK-2 cells; **(b)** The effect of HSYA on amount of ROS produced by HK-2 cells; **(c)** Changes of MDA production in HK-2 cells under different concentrations of HSYA supplementation; **(d)** Changes of SOD activity in HK-2 cells under different concentrations of HSYA supplementation; **(e)** Changes of activity of GSH-Px in HK-2 cells under different concentrations of HSYA supplementation; **(f)** The effect of different concentrations of HSYA on CAT activity in HK-2 cells. Compared with cells treated with CsA only, *p < 0.05 and **p < 0.01, compared with control, ^#^p < 0.05 and ^##^p < 0.01.

Compared with the CsA treat group, the cotreatment of HSYA and CsA could improve the viability of HK-2 cells. The cell viability increased with the increase of HSYA concentration, reaching the maximum value at 80 μmol·L^–1^ (Fig. 1a). At the same time, compared with the CsA alone group, ROS and MDA levels were down-regulated in a dose-dependent manner after treatment with HSYA (p < 0.05, Fig. 1b, c). In the CsA alone group, the ROS and MDA level was respectively 114.97 ± 6.76 and 52.01 ± 1.36. However, the results were respectively 69.87 ± 4.98 and 38.85 ± 1.22 in the 80 μmol·L^–1^ HSYA+CsA group (ROS concentration: kU·g^–1^, MDA concentration: nmol·L^–1^, p < 0.01, Fig. 1b, c). Also, the SOD activity of 80 μmol·L^–1^ HSYA+CsA group (32.6 ± 0.66) was higher than CsA alone group (21.88 ± 0.73) (kU·g^–1^ p < 0.05, Fig. 1d). In addition, compared with the CsA alone group, after being treated with 80 μmol·L^–1^ HSYA. The GSH-Px and CAT activity both increased with different extents (GSH-Px activity: 56.42 ± 2.32 vs 75.984 ± 3.07, (CAT activity: 1.56 ± 0.05 vs 1.83 ± 0.1) (kU·g^–1^, p < 0.05, Fig. 1e, f). The above studies showed that HSYA could reduce the increase of ROS and MDA caused by CsA, along with restoring the activities of SOD, GSH-Px, and CAT.

### HSYA inhibits apoptosis of HK-2 cells

**Figure 2:**
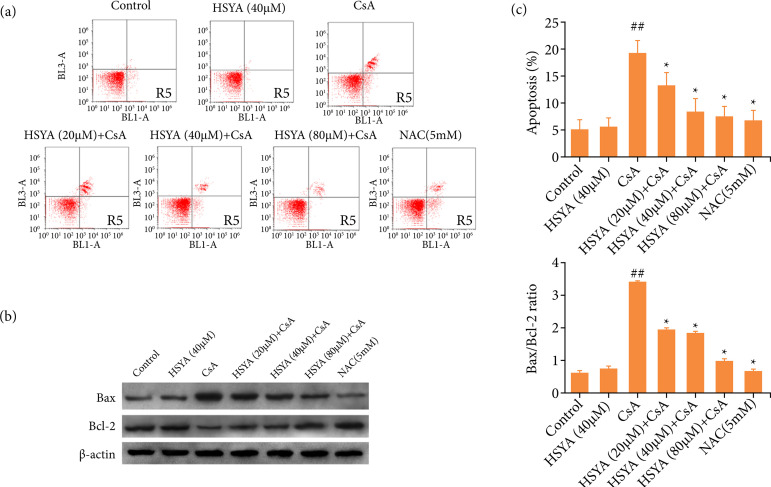
The effect of HSYA at different concentrations on HK-2 cell apoptosis and the expression of apoptosis-related proteins. **(a)** The effect of HSYA on apoptosis of HK-2 cell which was induced by CsA; **(b)** The effect of HSYA on the Bcl-2 and Bax protein expression levels in HK-2 cells; **(c)** Determine the effect of HSYA on the ratio of Bax/Bcl-2 in CsA-induced HK-2 cells by densitometric analysis. Compared with cells treated with CsA only, *p < 0.05 and **p < 0.01, compared with control, ^##^p < 0.01.

The results in Fig. 2a showed that with the increase of HSYA concentration, the apoptosis rate of HK-2 cells gradually decreased, which manifested that HSYA could inhibit CsA-induced apoptosis. Compared with the CsA alone group, when HSYA was co-treated with CsA, the expression of antiapoptotic protein Bcl-2 was upregulated, and the proapoptotic protein Bax was down-regulated. The ratio of Bax/Bcl-2 decreased (Fig. 2b, c). Therefore, HSYA could inhibit the apoptosis of HK-2 cells treated with CsA.

### HSYA alleviates CsA-induced renal tissue injury in rat CAN model

**Figure 3:**
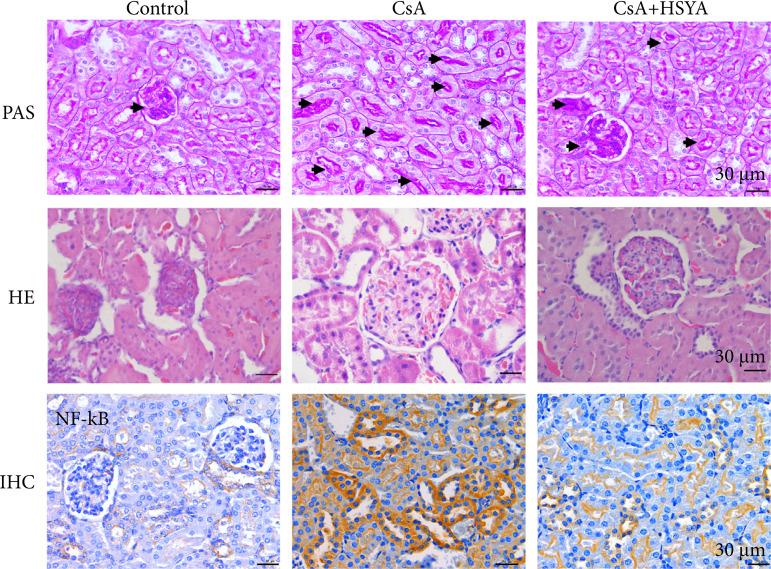
The effect of HSYA on the CsA-induced CAN rat model. Renal PAS staining showed that administration of HSYA reduced renal fibrosis in CsA-treated rats. Arrows indicate positive PAS staining. Kidney H&E staining showed that the structure of CsA treated rat kidney was improved after HSYA administration. Immunostaining of NF-κB indicated that HSYA reduced the inflammatory response in CsA-treated rats (PAS; NF-κB). The number of rats in each group is 6.

For further evaluation of the protective effect of HSYA in the rat model of nephrotoxicity induced by CsA, then the pathological features of rat renal tissue were examined. It was observed that the structure of tissue sections in the control group was normal by H&E staining (Fig. 3). On the contrary, there are noticeable histological changes such as cell edema, cell vacuole deformation and dissolution in the cell when the CsA induced the kidneys of rats. When CsA was combined with HSYA treatment, the tissue morphology was closer to the control group. In addition, PAS staining showed that the glomerular volume and hyperplasia of mesangial matrix (PAS positive), glomerular swelling, irregular shape of some glomeruli, lobulated capillary ring, and renal capsule space increased significantly in the CsA group, while HSYA could reduce CsA induced matrix proliferation. Simultaneously, the immunohistochemistry results showed that the control group, the NF-κB positive cells in the CsA group were higher than those in the control group, which suggested CsA plays a role in promoting inflammation. In contrast, the CsA and HSYA co-treatment group had fewer NF-κB positive cells. It showed that HSYA may reduce the occurrence of cell inflammation.

### HSYA improves renal function of CAN rats

**Figure 4:**
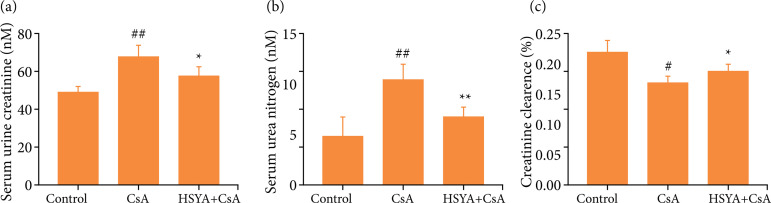
Effects of HSYA on the renal function indexes of rats induced by CsA. Compared with the group treated with CsA only, the number of rats in each group is 6. *p < 0.05, **p < 0.01; compared with control, ^#^p < 0.05, ^##^p < 0.01.

As shown in Fig. 4a–c, continuous administration of CsA (30 mg·kg^–1^), renal failure in rats 14 days later, manifested as a significant increase in serum urine Crea and urea nitrogen with the control group (p < 0.01), and a significant decrease in Ccr compared with the control group (p < 0.05). However, compared with the CsA group, BUN and Crea were significantly decreased (p < 0.05), and Ccr was increased significantly in HSYA treatment (p < 0.05). It meant HSYA could significantly improve nephrotoxicity caused by CsA.

## Discussion

As a powerful immunosuppressant, CsA can specifically act on lymphocytes, inhibit the synthesis and release of lymphokines (including IL-2), and block resting lymphocytes in the G0 phase and G1 early stage of the cell cycle. It has been shown that CsA inhibits cellular immunity to reduce the transplant rejection of allogeneic organ and tissue transplantation^12^. However, the dose-dependent nephrotoxicity caused by CsA was the main reason for its clinical application limitation. An earlier study confirmed that the increase in ROS caused by CsA leading to cell membrane lipid peroxidation is vital for its nephrotoxicity^13^. Oxygen-free radicals are the critical cause of ischemia-reperfusion injury. They can also directly induce the transcription and replication of interleukin-8 (IL-8), and accelerate the synthesis and release of IL-8^14^. Interleukin-8 could withstand the degradation of peptidase, make it accumulate and increase, and promote the production of oxygen free radicals, which in turn aggravates the inflammatory response and promotes the release of IL-8 and other cytokines, forming a vicious circle. Another study found that CsA could induce inducible nitric oxide synthase (iNOS) expression in the body, and the resulting high concentration of nitric oxide (NO) and ROS generate free radical activity^13^. It could alter the tricarboxylic acid cycle, mitochondrial function, electron transfer, which causes more pathological changes^15^. It has been suggested that oxidative stress may be involved in the pathogenesis of renal allograft fibrosis through its role in the epithelial-mesenchymal transition associated with CAN^13^. HSYA could inhibit nitric oxide synthase (NOS) activity, reduce the generation of free radicals induced by NO produced by the arginine metabolic pathway, thereby reducing its content in the kidneys and preventing NO toxicity^16^. This study confirmed that the effects of CsA included obviously upregulated MDA production and Bax expression and downregulated SOD activity and Bcl-2. It shows the same trend as the study by Huang *et al*.^17^. However, this study found HSYA inhibited the above CsA-induced cell damage and inhibited apoptosis.

The cell apoptosis in kidney tissue is mainly regulated by the Bax/Bcl-2 signaling pathway^18^. Bax has a proapoptotic effect, and Bcl-2 is an antiapoptotic protein. The ratio of Bax/Bcl-2 in the cell affects the occurrence of apoptosis^19^. HSYA has a good scavenging effect on oxygen-free radicals. As an excellent donor of hydrogen or neutrons, HSYA could react with excess free radicals generated by the organism in the oxidation-reduction reaction, thereby protecting the organism from free radical damage. Xie *et al*.^20^ found that HSYA can significantly antagonize the oxidative damage and apoptosis induced by H_2_O_2_. HSYA could reduce the inflammatory response induced by oxygen free radicals, reduce the changes in the integrity and permeability of the cell membrane caused by oxygen-free radicals damage to the cell membrane, and reduce the changes in membrane channels, ion pump functions, protein oxidation, DNA fragmentation and other cell apoptosis caused by damage. During cerebral ischemia and reperfusion, HSYA protects brain tissue by reducing oxidative stress damage in the rats’ brains^21^. At present, there was no report on the protective effect of HSYA in CsA-induced acute kidney injury and the protection of ischemia-reperfusion. This study confirmed that HSYA could significantly increase the antioxidant capacity of the kidneys and reduce the ratio of apoptosis in HK-2 cells treated with CsA. In addition, HSYA could alleviate CsA-induced renal failure, tubular deformation, and cellular vacuolization, thereby improving the function of the kidney.

In this research, continuous administration of CsA to rats showed significant nephrotoxicity, manifested as increased serum Crea and BUN levels, decreased Ccr, and a large number of cell vacuolar degeneration, disordered arrangement and renal tubular atrophy in kidney tissue. However, after HSYA treatment, the inflammatory factors in rat renal tissue were reduced, vacuoles between cells were reduced, and the renal function index was increased. The results above showed that HSYA was able to treat CsA-induced renal oxidative stress injury effectively. In addition, Hu *et al*.^22^ proved that HSYA could exert renal protection by inhibiting excessive apoptosis of renal tubular cells or enhancing the expression of apoptosis-regulating gene Bcl-2 protein. The development of kidney disease has previously been shown to be associated with NF-κB activation. Blocking the expression of NF-κB is one of the effective ways to reduce active oxidative metabolites, thereby improving the antioxidant capacity of the kidney^23^. NF-κB may also affect the inflammatory response in renal injury by modulating effects beyond the expression of inflammatory mediators. Therefore, it is closely associated with kidney diseases^24^. It has shown that inhibition of lysyl oxidase can reduce the progressive nephropathy and oxidative stress caused by CsA administration, thereby reducing the side effects of CsA^25^.

## Conclusion

The study shows the HSYA has a protective effect on CsA-induced nephrotoxicity. It is inferred that the mechanism may be related to the antioxidant capacity of HSYA.
